# Programme level implementation of malaria rapid diagnostic tests (RDTs) use: outcomes and cost of training health workers at lower level health care facilities in Uganda

**DOI:** 10.1186/1471-2458-12-291

**Published:** 2012-04-20

**Authors:** Daniel J Kyabayinze, Caroline Asiimwe, Damalie Nakanjako, Jane Nabakooza, Moses Bajabaite, Clare Strachan, James K Tibenderana, Jean Pierre Van Geetruyden

**Affiliations:** 1 Malaria Consortium, Upper Naguru East Road, P.O. Box 8045, Kampala, Uganda; 2 Foundation for Innovative New Diagnostics, Plot 23A Akii Bua Road, P.O. Box 34663, Kampala, Uganda; 3 Department of Medicine, Makerere University College of Health Sciences, P.O. Box 7072, Kampala, Uganda; 4 Malaria Control Programme, Ministry of Health, P.O. Box 7272, Kampala, Uganda; 5 Bugembe Health Centre IV, Jinja, Ministry of Health, P.O. Box 7272, Kampala, Uganda; 6 Unit International Health, Faculty of Medicine, Antwerp University, Universiteitsplein 1, Antwerpen, BE-2610, Belgium

## Abstract

**Background:**

The training of health workers in the use of malaria rapid diagnostic tests (RDTs) is an important component of a wider strategy to improve parasite-based malaria diagnosis at lower level health care facilities (LLHFs) where microscopy is not readily available for all patients with suspected malaria. This study describes the process and cost of training to attain competence of lower level health workers to perform malaria RDTs in a public health system setting in eastern Uganda.

**Methods:**

Health workers from 21 health facilities in Uganda were given a one-day central training on the use of RDTs in malaria case management, including practical skills on how to perform read and interpret the test results. Successful trainees subsequently integrated the use of RDTs into their routine care for febrile patients at their LLHFs and transferred their acquired skills to colleagues (cascade training model). A cross-sectional evaluation of the health workers’ competence in performing RDTs was conducted six weeks following the training, incorporating observation, in-depth interviews with health workers and the review of health facility records relating to tests offered and antimalarial drug (AMD) prescriptions pre and post training. The direct costs relating to the training processes were also documented.

**Results:**

Overall, 135 health workers were trained including 63 (47%) nursing assistants, a group of care providers without formal medical training. All trainees passed the post-training concordance test with ≥ 80% except 12 that required re-training. Six weeks after the one-day training, 51/64 (80%) of the health workers accurately performed the critical steps in performing the RDT. The performance was similar among the 10 (16%) participants who were peer-trained by their trained colleagues. Only 9 (14%) did not draw the appropriate amount of blood using pipette. The average cost of the one-day training was US$ 101 (range $92-$112), with the main cost drivers being trainee travel and per-diems. Health workers offered RDTs to 76% of febrile patients and AMD prescriptions reduced by 37% six weeks post-training.

**Conclusion:**

One-day training on the use of RDTs successfully provided adequate skill and competency among health workers to perform RDTs in fever case management at LLHF in a Uganda setting. The cost averaged at US$101 per health worker trained, with the main cost drivers being trainee travel and per diems. Given the good peer training noted in this study, there is need to explore the cost-effectiveness of a cascade training model for large scale implementation of RDTs.

## Background

With the widespread implementation of the use of artemisinin-based combination therapies (ACTs) as first-line anti-malarials in many African countries [1], World Health Organisation (WHO) recommends parasite-based testing for all suspected malaria cases prior to treatment [2] in order to reduce drug wastage, as a result of the unnecessary use ACTs, delay the spread of drug resistance, and to promote appropriate treatment for alternative causes of fever. Whereas microscopy is the main malaria diagnostic tool in many African countries, a lack of the required infrastructure and/or trained laboratory personnel remains a major challenge, particularly in lower level health facilities. There is a need to explore the use of malaria rapid diagnostic tests (RDTs) to be performed by various health care cadres after relatively minimal training, which would enable rapid scale up over large areas. Training of health workers and the provision of simple diagnostic tools have been identified as potential strategies to improve malaria case management and encourage the rational prescription of anti-malarial drugs (AMD) [3,4].

Task shifting is becoming a popular strategy to improve health care within the constraints of limited resources in sub Saharan Africa [5-7] and may improve malaria case management if parasitological diagnosis is not limited to laboratory technicians. RDTs may be performed by lower level laboratory/clinical personnel [8-12] and may therefore be incorporated into routine patient consultations to enhance clinical diagnosis and tailor malaria treatment to test results. RDTs are easy to use, require less skill to perform correctly and can provide accurate diagnosis at lower level health care facilities (LLHFs) in the absence of microscopy [13-15]. The Uganda malaria treatment policy was revised in 2009 to incorporate the use of RDTs for parasite-based malaria case management at LLHFs [16]. However, there is limited documentation on the implementation steps, such as the training process, to attain the competence to perform, interpret and utilise RDT test results among lower-level health workers. In order to gain operational experience to inform policy, this project provided training in RDTs use and introduced them at LLHFs in Uganda.

This implementation research was conducted to train and document the process of training health workers to perform and use RDTs in a public health care setting. Observations from this study should guide the National Malaria Control Programme (NMCP) in the training and scale up of RDTs use as one of the strategies to implement the policy of parasite-based diagnosis and treatment of malaria in the public sector.

## Methods

### Study design and setting

In a cross-sectional study, one-day training on the use of RDTs in malaria case management was conducted for health workers at LLHFs, typically primary health facilities that serve 1,000-25,000 people (Health Centre II level), within five eastern districts in Uganda. Each district was a representative of the different malaria epidemiological settings in Uganda; that is; Kapchorwa - a hypo-endemic, Mudende - meso-endemic and Iganga – a hyper endemic setting. Jinja and Mbale were included to represent the fourth epidemiological stratum (urban setting), which potentially differs from the rural settings in terms of knowledge, health seeking behaviour and general health services delivery. The one-day training was done within a larger implementation trial to determine the utility of RDTs at LLHFs [17].

One week following the one-day training, RDTs (P.f™ ICT, manufactured by ICT Diagnostics, South Africa) were distributed to all the study health care facilities together with accessories, including gloves, sharps disposal bins, lancets and timers. The facilities were eligible for training on RDTs if they met the following criteria: 1) current lack of functional parasite-based diagnostic services, 2) no previous involvement in a similar research, 3) over 200 suspected malaria cases managed at the facility per week and 4) availability of a recording system that comprised outpatient registers, health management information system (HMIS) and drug consumption data. Of the eligible facilities (Kapchorwa 10, Jinja 10, Iganga 15, Mbale 12, and Mubende 11), four were randomly selected from each district. In each of the five districts, one health facility did not receive training and was used as a comparison group. Baseline data was extracted from out-patient department (OPD) registers to document antimalarial prescriptions practices before the training and introduction of RDTs.

### Training and evaluation of health workers on the day of training

**Figure 1:**
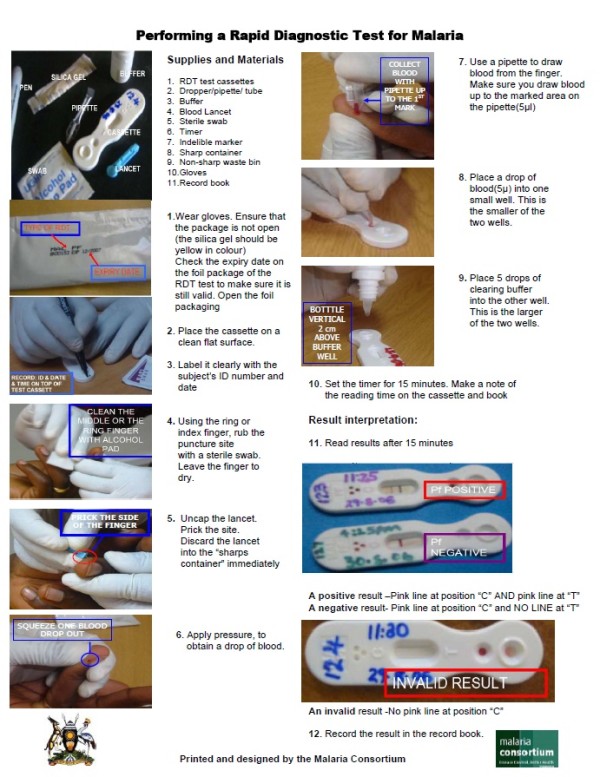
**Study Job Aid: How to perform a malaria RDT.** The pictorial job aide shows the procedure for performing a RDT for malaria. The chart is used to train and to evaluate health workers’ ability to accurately perform a test. All the steps must be performed correctly or the result may be incorrect and the result must be interpreted correctly or diagnosis may be incorrect. This job aide can be used with little supervision at peripheral levels

All health workers from the selected facilities were invited to a district-based training in the use of RDTs for malaria in a staggered fashion to ensure minimal interruption of the service delivery at the health care facilities. Two one-day trainings were conducted, each consisting of half the trainees with a ratio of 5:1 trainees for one facilitator. The training and assessment tools were piloted and standardized in accordance with the Ministry of Health. A total of 135 health workers received training. A pre-training exercise was conducted to assess the health workers’ knowledge in malaria case management. The training was based on the WHO curriculum for the use of RDTs [17] and the content included concepts and guidelines of parasite-based management of malaria, related record keeping, distribution, storage and waste disposal of RDTs, a RDT job aide, and guidance in hands-on practice in performing a RDT. After the training, the pre-training test exercise was repeated to assess the knowledge gained. In addition, health workers were observed and scored for adherence to the procedures to perform the RDTs based on the pictorial chart used during the training (see Figure 1).

Participants were considered competent if they scored ≥ 80% in the post-training practical concordance test (see Additional file 1: Figure S1). Participants that scored < 80% were retrained the following day. Subsequently, all the health workers were allowed to practice, perform and use the RDTs in the selected health facilities. Health workers were trained to treat according to RDTs test results. However, they still had liberty to use presumptive treatment for children under five years given this was the guidance provided in the national malaria control policy at the time of the study. Health workers who received the training were encouraged to train other health workers in their respective health care facilities (cascade model training).

### Post-training evaluation after six weeks

Six weeks after the one-day training, health workers were observed and evaluated for competence in performing RDTs in the out-patient clinics on a normal working day. The sample included all health workers who had received training on RDT use, were on duty on the day of the evaluation and who had given written informed consent to participate in the study. In addition, we observed the health workers who had received only on-job training by their colleagues. The health workers were assessed for performance of the RDT, reading and interpretation of results. Scores were awarded by the trainers for every step performed correctly (see Additional file 2: Table S1). In-depth interviews were also conducted with each health worker to explore their perceptions on ease of use, willingness to perform the test and interpret the results, and feasibility of performing RDTs as part of routine management of suspected malaria cases. Proportions of febrile patients offered a test and proportion prescribed AMDs depending on RDT results were calculated from OPD register books.

### Cost of the one-day training intervention

All trainees were provided with a transport refund, per diem and one night’s accommodation on the day of the training. The data on direct training costs were collected retrospectively from financial and operational records, in particular expenditure and salary records. Additional costing information was acquired from interviews with key personnel; in particular the project’s Logistic Manager Data extraction was based on an itemised menu approach (Drummond MF 2005 Oxford press) and was undertaken between July and December 2008 at the time of the parent study. The results are presented from the implementer’s perspective. Where possible both financial and economic costs were collected in order to estimate financial requirements for programme implementation. The financial costs included monetary costs of the training while economic costs represent the value (or opportunity cost) of the resources used to implement the training including the trainees’ time as estimated from government pay scale. Research costs were not included except support supervision after the training which we considered as a recurrent cost in large scale implementation of RDTs. Operational costs included venue hire, meals and transportation of trainees to the practical training sites, transport and per diem, lodging and incidental expenses for both trainers and trainees. Training materials were not included in this estimate given they are readily available from the NMCP and capital costs were not included given the short term nature of the training. Programme implementation costs were classified into a) set-up costs which included a three-day trainer of trainers (TOT) based on a pre-developed protocol/guidelines and b) recurrent costs such as the one-day training (recurrent because of the need for refresher training of health workers given the high rates of staff turnover) as well as the national and local supervision costs. The cost of training each health worker including the follow up supervision was computed based on expense documents. The ingredient approach was used, whereby inputs were identified valued and classified into activities. Where this approach was deemed too sensitive (salaries and overhead costs), aggregated expenditure was used. All costs were converted to United States dollars (USD) based on official yearly average exchange from OANDA (http://www.oanda.com/convert/classic assessed on 20/11/2011).

### Ethical considerations

The study was conducted according to the principles of the declaration of Helsinki and the international guidelines of biomedical research involving human subjects (http://www.cioms.ch/frame_guidelines_nov_2002.htm). Ethical approval was granted by the Uganda National Council for Science and Technology (UNCST). Support for the study was acquired from the ‘in-charge’ of each of the selected health facility and each participant provided written informed consent to participate.

### Data management and analysis

Quantitative data were double entered into the study database using a data-entry template in EpiData (“The EpiData Association” Odense, Denmark). The two data files were compared to check for completeness and conflicting entries. One copy was then cleaned and exported to SPSS 12 (SPSS USA 2005) for further checks on completeness of the data and subsequent analysis.

Demographic data were recorded for the health workers that attended the training and the subsequent evaluation of competences. The skills and competences attained were compared among the nursing assistants (who have limited formal training) and the formally trained health workers (nurses, clinical officers and laboratory technologists) to evaluate the feasibility of transferring the skills of performing RDTs to the lowest cadre of health care providers at LLHFs. Qualitative data from in-depth interviews of health workers was analysed manually using pre-set themes of ease of use of the test, competence and confidence to perform the test and feasibility of using RDTs in routine management of suspected malaria. Costing information was documented and analysed.

## Results

### Health worker training

A total of 135 health workers were trained within the 5 districts of Iganga, Jinja, Kapchorwa, Mbale and Mubende. Each district was represented by health workers from at least four health care facilities. The health workers included clinical officers, nurses, laboratory technicians and nursing assistants (who are usually trained on the job through apprenticeships); see Table 1. The majority, 65/135, (48%) of the health workers trained were nursing assistants. On average all health workers had comparable scores in the pre- training test (mean 59%, SD 20.16, p < 0.01). Out of the 128 trainees with both pre and post-training results, the post-training mean score of 77% (SD 16.9) was notably higher than pre-test score (paired t-test value =32.7, p < 0.01). The post-training test score for nursing assistants was notably lower 71% (SD 19%) compared to the professional health workers at 80% (SD 14%) (p = 0.003). All trainees passed the post-training practical concordance test with ≥ 80% except 12 (9%) who required re-training to further master the practical skills. The trainees who had higher post-training test scores generally scored higher in the practical concordance test (Spearman’s correlation coefficient 0.3889, p = 0.03).

**Table 1:** Health worker (HW) cadres at the lower level health care facilities at the 5 districts in Uganda who participated in the training on RDT use

**Characteristics**	**Iganga**	**Jinja**	**Kapchorwa**	**Mbale**	**Mubende**	**Overall (%)**
Health facilities	4	4	4	5	4	21
**Health worker cadres**						
Clinical officers	2	0	2	2	2	8(6%)
Nurses	12	3	12	12	9	48(36%)
Laboratory technicians¥	0	0	0	2	0	2(1%)
Nursing Assistants∏	11	7	13	24	11	65(48%)
**Total number of HWs trained**	**25**	**10**	**27**	**51**	**22**	**135**

¥ Lower level health care facilities in Mbale did not have facilities to perform microscopy although the laboratory technicians were available.

∏These include vaccinators

### Competences of health workers in performing RDT tests

A total of 64(47%) health workers were available for the six weeks post training evaluation. During the evaluation, 51/64 (80%) of all health workers accurately performed the steps critical for performing a RDT, and interpreted and recorded the results correctly in the books and registers (see Table 2). Only 10/64 (16%) of the health workers did not draw the appropriate amount of blood using the pipette. Out of 14,940 patients who presented with fever during the six weeks post training, 11,354 (76%) were offered a RDT for malaria. The antimalarial drug prescription reduced from 8432/14024 (56%) to 4892/14940 (33%) over the same period.

**Table 2:** Observed health worker competence in performing RDTs at their health facilities six weeks post-training

	**Competences assessed**	**Nursing Assistant s = 26(%)**	Professional Health workers*n = 32(%)
**Preparation**	Places cassette on flat surface	22(88)	32(100)
	Identifies patient with clinic file number	22(88)	30(94)
**Performs test**	Cleans finger with alcohol cotton swab	23(89)	32(100)
	Uses sterile lancet to puncture (uses lancet only once.	22(88)	32(100)
	Collects appropriate amount of blood with device	18(69)	28(88)
	Places blood in appropriate well	22(88)	29(91)
	Holds bottle vertically to dispense buffer	21(84)	28(88)
	Dispenses correct amount of buffer (5 drops)	19(79)	31(97)
**Results Interpretation**	Reads result within correct period of time	21(84)	32(100)
	Reads results correctly	19(82)	31(97)
	Interprets and records results correctly	20(83)	31(97)
**Safe waste disposal**	Disposes of used materials correctly	20(83)	32(100)

Nursing assistants are trained on the job through apprenticeship

*Professional health workers include: 22 enrolled nurses/midwives, 4 laboratory technicians and 4 clinical officers

*Ten health workers were not centrally trained, but learnt on-the-job from trained colleagues and their performance was comparable

### Resources used to deliver the one-day training on RDT use

The average cost of the one-day training per person trained was $101 (range $92-$112). Over half of the total cost of training $7,861/15,160 (52%) was spent on participants’ travel and per-diem (see Table 3). An additional benefit was the cascade model training of 10 participants that were trained by peers however; this incidental finding was not included in the analysis.

**Table 3:** Resources used to implement five ‘one-day training’ in the use of RDTs at lower-level health care facilities at 5 districts in Uganda

**Human resources**		**Cost of training health workers to perform malaria RDT**		
**Cadres of trainers**∏ **N = 6**	**Cadres of trainees∑ N = 135**	**Cost Item**	**Cost (USD)**	**Average cost per individual trained (USD)**
Training coordinator	Clinical officers	Personnel	$2,582	$101 (range 92–112)*
Clinical epidemiologist	Nurses	Training Supplies	$554	
Medical doctor	Nursing assistants	Operational costs	$2,785	
Laboratory technologist	Vaccinators	Travel and subsistence	$7,861	
Administrator	Records officers	Administrative costs	$1,378	
	Malaria focal person			
	Facility volunteers	Total	$15,160	

**∑** Health care facility was represented at least 4 health workers

∏ Trainer: trainee ratio at each practical session was 1:5

*Range reflects the difference in the number of trainees planned for versus the number that showed up especially for the standard costs such as venue, transport that are independent of the numbers

### Ease of use of RDTs

During the interviews, all health workers reported that they felt able to perform all the steps in making a malaria diagnosis using RDT and were competent to perform the test independently. There was no notable difference between the reported ease among the nursing assistants and the formally qualified health staff (professional health workers). Five (20%) nursing assistants reported difficulty in pricking a finger and filling a pipette with blood as the most challenging steps. All the other steps were well performed considering that over 85% reported them as very easy to perform tasks.

### Health workers’ perceptions on use of diagnostics

During the follow up evaluation, the health workers reported that training materials were easy to use and many of them felt they were able to train other health workers. In addition, majority (98%) of the health workers felt that they had acquired adequate knowledge and skills to integrate RDTs into their routine patient care. However some health workers (50%) felt that there is need to increase the numbers of health workers if RDTs are to be performed regularly.

## Discussion

Our study showed that health workers across different cadres and with different levels of pre-service training were able to learn how to effectively perform the ICT RDT, from a one day training session. Nursing assistants experienced some difficulties in pricking the finger with a lancet and filling a pipette with blood. Similar difficulties have been reported in Tanzania [18], South Africa and Zambia [19,20] among low level cadres although these are generally overcome with practice. Apart from the practical skill of performing the RDT, it was realised that the informally trained health workers (nursing assistants) require additional training to improve their knowledge on aspects of prevention and specific treatment of malaria and non-malaria fever cases. These results imply a potentially important role of RDTs in the implementation of parasite-based malaria diagnosis by health workers at LLHFs in Uganda where trained laboratory technicians or the infrastructure to support routine microscopy are not readily available.

The one-day training given at a central location seems sufficient to transfer the skills necessary to roll-out the use of RDTs for malaria diagnosis at peripheral health facilities in Uganda. Immediate utilisation of RDTs (within 6 weeks of training) appears to be recommended as evidenced by the finding that 75% of febrile patients who presented to the health facilities thereafter were offered a diagnostic test. It is important to design studies to evaluate the long-term retention of knowledge and skills attained as well as to assess the need and timing of re-fresher training to maintain the high utilization and quality in performance of RDTs for parasite-based malaria diagnosis at lower level health care facilities.

It was interesting to demonstrate that health workers who received the one-day training in RDT use were able to multiply this effect by adequately training their counterparts at their respective health facilities (cascade model training). This finding is consistent with reports from the malaria case-management team at the Infectious Diseases Institute in Uganda, which showed that cascade training by trainees after a trainer of trainers (TOT) course was comparable to the initial first-hand training [21]. However, this was an incidental finding in our study and there is not enough data to compare it with the one-day first-hand training. There is a need to further explore this strategy given the fact that over half of the training costs were spent on participants’ transport and per-diem. Peer training may be a potential mechanism to maintain use of RDTs in the context of high health worker turn over in addition to the fact that periodic training may be limited after country-wide implementation of RDTs. Future training programmes should systematically evaluate the utility and cost-effectiveness of the cascade training model in the implementation of parasite-based malaria diagnosis.

This is one of the first studies to document the cost of the training process on the use of RDTs in a resource-limited setting. The one-day training cost up to US $101 to train one health worker to use RDTs. The documented costs included the cost of facilitating the core trainers from the centre (Ministry of Health and Malaria Consortium) to the districts, transport and operational costs. This study did not document capital costs including preparation of training materials since this is an intervention that should be integrated into the current health care system to improve the efficiency of service delivery. We recommend cost-effectiveness studies to understand the utility and long-term impact of one-day on the scale up of parasite-based malaria case management in resource-limited settings.

Lessons learnt: Given the limited numbers of health workers, there is need for strategic phasing of the training sessions to avoid interruption of services at the health facilities. It is for this reason that we performed two one-day trainings to have half the trainees attend independently while the other half offered patient care and vise-versa. Within the context of the health system where the majority of health care providers are not formally trained, the training content should be tailored to suit the lower academic levels and should include more on background knowledge in addition to the practical skills.

### Limitations

This study did not cost the preparation of the training materials since these materials are readily available from the NMCP. Generic training materials have also been made available through collaborations with international agencies like WHO [22]. It is likely that the observed performance could have been biased since the health workers were aware that they were being observed and scored. This could amounts to a Hawthorne effect, but it does not affect the outcomes because it was the same bias introduced during the two assessments; during the training and six weeks post-training. In addition, the study design did not include health facilities to receiving RDTs without training as a comparative arm to facilities with training + RDTs. Such a design would not be feasible for an operational study. Therefore it was not possible to attribute the antimalarial saved to the training alone, or even extrapolate the impact in this setting. Routine health facility data was used to document patients tested without being able to quality control its acquisition. However the main outcome variables of training were collected by our study terms.

## Conclusion

One-day training cost $101 per health worker and successfully delivered adequate skills and competences among health workers to perform and use RDTs in fever-case management at LLHFs in Uganda. The cascade training model should be explored as a potential strategy for affordable implementation and scale up of parasite-based diagnosis of fever at LLHCFs in Uganda.

## Competing interests

The authors declare that they have no competing interests.

## Authors’ contributions

All authors read and approved the final manuscript. DJK CA and JKT participated in the conception and design of the study, protocol development and the training, and participated in data analysis and interpretation of results. DJK and DN wrote the first draft of the manuscript and were responsible for finalising this article. CA coordinated the study. CS participated in manuscript review. JN and MB participated in the training of health workers and acquisition of data. JKT and JPV provided technical oversight, critical review of the manuscript and its revisions.

## Pre-publication history

The pre-publication history for this paper can be accessed here:

http://www.biomedcentral.com/1471-2458/12/291/prepub

## Supplementary Material

Additional file 1 Figure S1. Scatter plot showing the concordance test results plotted against post test written results for 126 trainees that participated in the study to introduce RDTs at lower level health centres in Uganda 2007. Click here for file

Additional file 2 **Table S1. Date collection instrument used to evaluate Health worker performing RDT with instructions to the supervisor.** During the post - RDT training supervisory visit, observe and assess the performance of each step for a maximum of five RDTs performed by each user. All problems encountered by the user which should be addressed during subsequent discussions. Click here for file
