# Supplementary material for: Programme level implementation of malaria rapid diagnostic tests (RDTs) use: outcomes and cost of training health workers at lower level health care facilities in Uganda
Source: BMC Public Health. 2012 Apr 20;12:291. doi: 10.1186/1471-2458-12-291 (PMC3433367; doi:10.1186/1471-2458-12-291)

Additional figure 1: Scatter plot showing the concordance test results plotted against post test written results for 126 trainees that participated in the study to introduce RDTs at lower level health centres in Uganda 2007


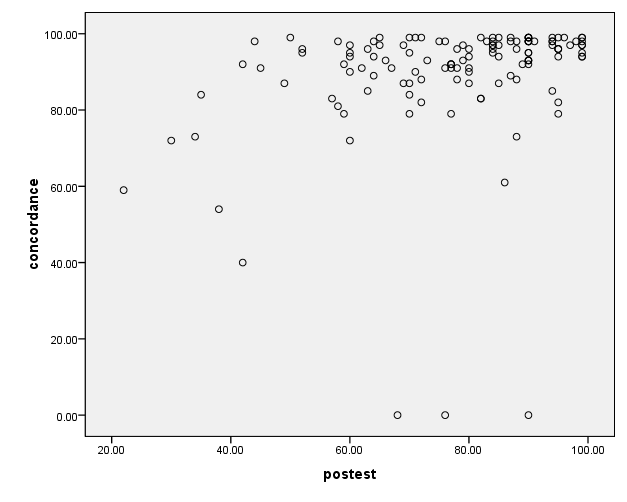

Supplement: Additional file 1 — Figure S1. Scatter plot showing the concordance test results plotted against post test written results for 126 trainees that participated in the study to introduce RDTs at lower level health centres in Uganda 2007. [file 1471-2458-12-291-S1.docx]
