# Supplementary material for: Programme level implementation of malaria rapid diagnostic tests (RDTs) use: outcomes and cost of training health workers at lower level health care facilities in Uganda
Source: BMC Public Health. 2012 Apr 20;12:291. doi: 10.1186/1471-2458-12-291 (PMC3433367; doi:10.1186/1471-2458-12-291)
Supplement: Additional file 2 — Table S1. Date collection instrument used to evaluate Health worker performing RDT with instructions to the supervisor. During the post - RDT training supervisory visit, observe and assess the performance of each step for a maximum of five RDTs performed by each user. All problems encountered by the user which should be addressed during subsequent discussions. [file 1471-2458-12-291-S2.docx]

Additional file 1: Data collection instrument Instructions for supervisor:

During the post - RDT training supervisory visit, observe and assess the performance of each step for a maximum of five RDTs performed by each user. All problems encountered by the user which should be addressed during subsequent discussions

|  | **Award a Yes (Y) or No (N)** | **Number of RDTs performed** | | | | |
| --- | --- | --- | --- | --- | --- | --- |
| 1 | Prepares working space in well lit and safe area |  |  |  |  |  |
|  |  | 1 | 2 | 3 | 4 | 5 |
| 2 | Refers to RDT job aid for steps below; |  |  |  |  |  |
| 3 | Checks to ensure RDT packaging is undamaged |  |  |  |  |  |
|  |  |  |  |  |  |  |
| 4 | Places RDT on flat surface in a recommended position |  |  |  |  |  |
|  |  |  |  |  |  |  |
| 5 | Records patient data on RDT cassette/strip |  |  |  |  |  |
|  |  |  |  |  |  |  |
| 6 | Selects correct hand and finger (3rd) to puncture and without sores |  |  |  |  |  |
|  |  |  |  |  |  |  |
| 7 | Cleans finger with alcohol cotton swab. |  |  |  |  |  |
|  |  |  |  |  |  |  |
| 8 | Uses sterile lancet to puncture (use lancet only once) and punctures (not drill) at correct position |  |  |  |  |  |
|  |  |  |  |  |  |  |
| 9 | Collects appropriate amount of blood with device (e.g 10µl) |  |  |  |  |  |
|  |  |  |  |  |  |  |
| 10 | Places blood in appropriate well of RDT |  |  |  |  |  |
|  |  |  |  |  |  |  |
| 11 | Holds bottle in vertical to dispense buffer |  |  |  |  |  |
|  |  |  |  |  |  |  |
| 12 | Dispenses correct amount of buffer (refer to job aid) |  |  |  |  |  |
|  |  |  |  |  |  |  |
| 13 | Reads test result within correct period of time |  |  |  |  |  |
|  |  |  |  |  |  |  |
| 13 | Reads result correctly (*this step should be practiced using RDT result quiz photographs*) |  |  |  |  |  |
|  |  |  |  |  |  |  |
| 14 | Interprets and records results correctly |  |  |  |  |  |
|  |  |  |  |  |  |  |
| 15 | Disposes of used materials correctly |  |  |  |  |  |
|  |  |  |  |  |  |  |
|  | TOTAL for each test performed |  |  |  |  |  |

### TOTAL: _____/75 points maximum

*(Scoring: For each YES– 1 point, for each NO – 0 points)*

Observations on RDT test performance
